# Cost-Effectiveness of Bronchial Thermoplasty, Omalizumab, and Standard Therapy for Moderate-to-Severe Allergic Asthma

**DOI:** 10.1371/journal.pone.0146003

**Published:** 2016-01-11

**Authors:** Zafar Zafari, Mohsen Sadatsafavi, Carlo A. Marra, Wenjia Chen, J. Mark FitzGerald

**Affiliations:** 1 Institute for Heart and Lung Health (IHLH), Faculty of Medicine, University of British Columbia, Vancouver, Canada; 2 Center for Clinical Epidemiology and Evaluation, Vancouver Coastal Health Institute, University of British Columbia, Vancouver, Canada; 3 Respiratory Medicine Division, Faculty of Medicine, University of British Columbia, Vancouver, Canada; 4 School of Pharmacy, Memorial University of Newfoundland, St. John's, Canada; 5 Collaboration for Outcomes Research and Evaluation, Faculty of Pharmaceutical Sciences, University of British Columbia, Vancouver, Canada; National Institute for Public Health and the Environment, NETHERLANDS

## Abstract

**Background:**

Bronchial thermoplasty (BT) is a recently developed treatment for patients with moderate-to-severe asthma. A few studies have suggested the clinical efficacy of this intervention. However, no study has evaluated the cost-effectiveness of BT compared to other alternative treatments for moderate-to-severe allergic asthma, which currently include omalizumab and standard therapy.

**Objective:**

To evaluate the cost-effectiveness of standard therapy, BT, and omalizumab for moderate-to-severe allergic asthma in the USA.

**Methods:**

A probabilistic Markov model with weekly cycles was developed to reflect the course of asthma progression over a 5-year time horizon. The study population was adults with moderate-to-severe allergic asthma whose asthma remained uncontrolled despite using high-dose inhaled corticosteroids (ICS, with or without long-acting beta-agonists [LABA]). A perspective of the health-care system was adopted with asthma-related costs as well as quality-adjusted life years (QALYs) and exacerbations as the outcomes.

**Results:**

For standard therapy, BT, and omalizumab, the discounted 5-year costs and QALYs were $15,400 and 3.08, $28,100 and 3.24, and $117,000 and 3.26, respectively. The incremental cost-effectiveness ratio (ICER) of BT versus standard therapy and omalizumab versus BT was $78,700/QALY and $3.86 million/QALY, respectively. At the willingness-to-pay (WTP) of $50,000/QALY and $100,000/QALY, the probability of BT being cost-effective was 9%, and 67%, respectively. The corresponding expected value of perfect information (EVPI) was $155 and $1,530 per individual at these thresholds. In sensitivity analyses, increasing the costs of BT from $14,900 to $30,000 increased its ICER relative to standard therapy to $178,000/QALY, and decreased the ICER of omalizumab relative to BT to $3.06 million/QALY. Reducing the costs of omalizumab by 25% decreased its ICER relative to BT by 29%.

**Conclusions:**

Based on the available evidence, our study suggests that there is more than 60% chance that BT becomes cost-effective relative to omalizumab and standard therapy at the WTP of $100,000/QALY in patients with moderate-to-severe allergic asthma. However, there is a substantial uncertainty in the underlying evidence, indicating the need for future research towards reducing such uncertainty.

## Introduction

Asthma is a common chronic inflammatory disease of the airways with a substantial global burden in terms of costs, morbidity, and reduced quality of life [1]. Compared with typical patients with asthma, patients with moderate-to-severe asthma generally have significant impairment and consume more health-care resources, especially if their asthma is not controlled [2]. While inhaled corticosteroids (ICS) are the mainstay of asthma therapy, some patients with moderate-to-severe asthma do not achieve control even with high dose ICS [3,4]. In such patients, the addition of a long-acting beta-agonist (LABA) can further improve asthma control but a significant proportion would still remain uncontrolled. There have been some promising developments in terms of new therapeutic options for this subgroup of patients. Omalizumab, which is a humanized monoclonal antibody targeting the IgE, is the first of likely many new biologics available for the treatment of moderate-to-severe allergic asthma, which is limited to atopic subjects with an elevated IgE level within a fairly narrow range [5].

Recently, another approach to the treatment of this patient population has been proposed. Bronchial thermoplasty (BT) is a technique whereby radiofrequency ablation is applied sequentially to the peripheral sub-segmental airways. The procedure involves three bronchoscopies during which sequentially segmental airways are treated [6]. Two randomized controlled trials have shown that BT reduces the rate of asthma exacerbations compared with standard (ICS+LABA) therapy [7,8]. A recent follow-up study has provided some evidence regarding safety and ongoing benefits of BT up to five years after the procedure [9]. Nevertheless, there is still uncertainty about BT’s long-term health benefits, indicating a need for further studies. In contrast, omalizumab has been shown to have a substantial impact on reducing the number of exacerbations and improving quality of life [10]. However, its cost-effectiveness versus BT has not yet been studied.

The purpose of this study was to evaluate the cost-effectiveness of omalizumab, BT, and standard therapy over a five year time-horizon in patients with moderate-to-severe allergic asthma in the U.S. We hypothesized that the health-related outcomes at the population level can be improved, while health-related costs can be decreased when using BT compared with standard therapy and omalizumab.

## Methods

A probabilistic decision-analytic Markov model was developed to compare the economic and humanistic burden associated with standard therapy, BT, and omalizumab in individuals with moderate-to-severe allergic asthma who remain uncontrolled despite using high dose ICS or ICS+LABA. Since the effect size of BT was informed from studies with, at most, a 5-year follow-up and due to a lack of evidence for the continued efficacy for a longer duration [9], we considered 5 years as the time horizon of the base case analysis. We also reported the cost-effectiveness outcomes over a life time, in which we conservatively, assigned a declining effect to BT’s efficacy after the fifth year. In addition, we investigated other time horizons (i.e., 10 years with or without declining effect for BT after the fifth year, and 5 years with a declining effect for BT after the first year) in the sensitivity analyses. Standard therapy was defined by the Steps 3 and 4 of the Global Initiative for Asthma (GINA) treatment strategy [11]. All patients were adults between 18 and 65 years old with a mean age of 40 years which is similar to the previous studies evaluating the effectiveness of BT [7]. At baseline, all patients were uncontrolled despite using high-dose (≥ 1,000 mcg of fluticasone or equivalent) ICS. Short-acting beta-agonists (SABA) were presumed to be used as a reliever medication by all patients. The primary outcomes of the analysis were the discounted direct costs, discounted quality-adjusted life years (QALYs), and the corresponding incremental cost-effectiveness ratio (ICER) over the 5-year time horizon. ICERs were evaluated at two willingness to pay (WTP) thresholds of $50,000/QALY and $100,000/QALY [12–14]. The secondary outcomes were the total number of asthma exacerbations and the proportion of subjects who died. The analysis was from the health-care system perspective and future outcomes were discounted at 3% [15,16].

### Model Structure

**Fig 1:**
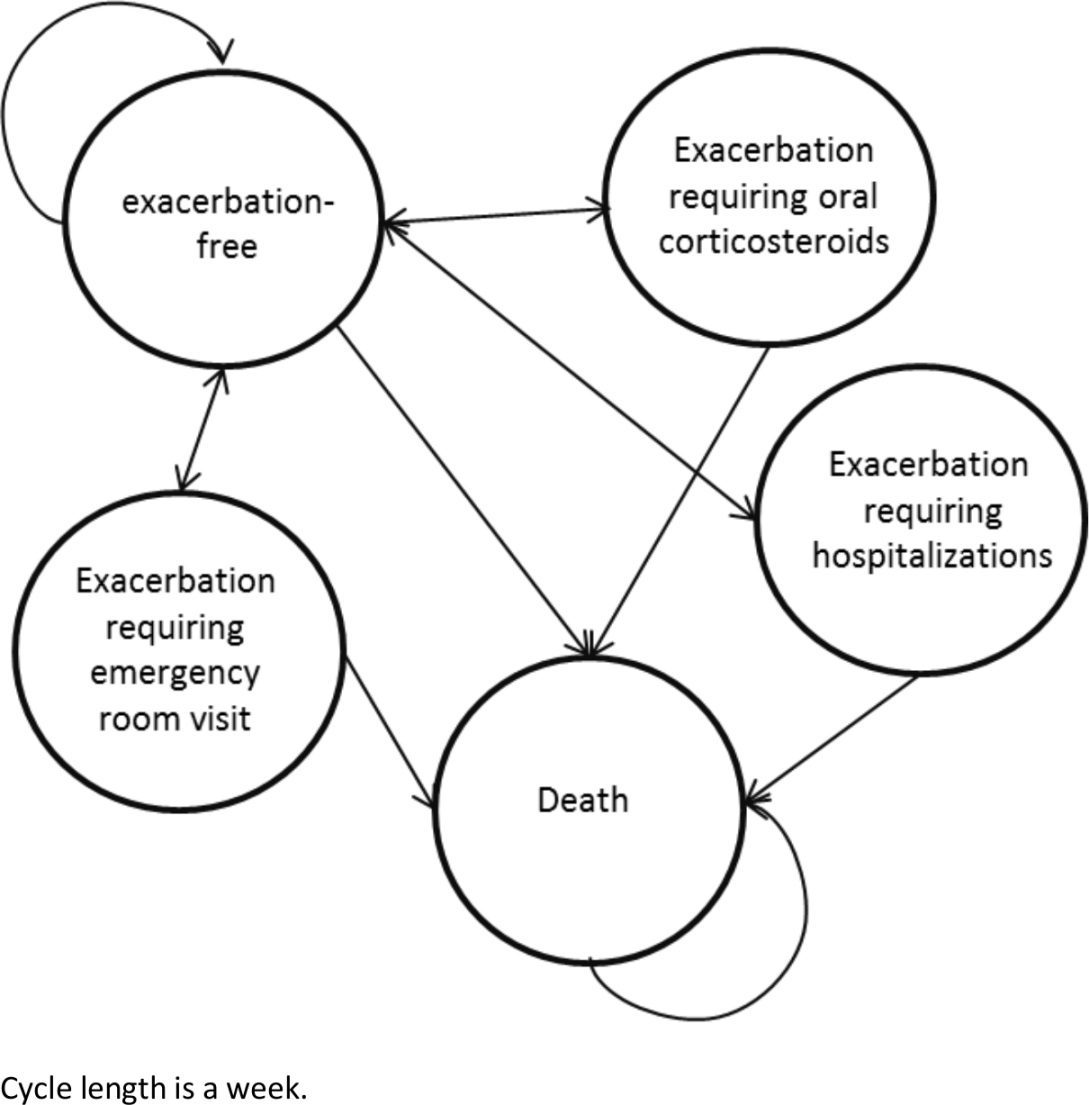
Schematic Markov states. Health states include: exacerbation-free, exacerbation states: exacerbation requiring oral corticosteroids, emergency room visit, hospitalizations, and death state.

A probabilistic discrete-time Markov model with five health states was used for this analysis. The structure of the model is informed from previous studies [17–23]. In addition to an absorbing state representing death, we modeled four discrete asthma-related states: exacerbation-free and the following three exacerbation states: 1) requiring oral corticosteroids (OCS); 2) requiring a visit to the Emergency Department [ED]; and 3) requiring hospitalization. A schematic illustration of the model is provided in Fig 1. The time cycle of the model was chosen as a week to allow modeling of exacerbation as an independent health state [24]. We used the statistical program R 3.1.0 to develop and run the model [25].

### Model Parameters

***Table 1***contains all the model parameters including exacerbation rates for interventions, costs of exacerbations (requiring OCS, ED, or hospitalization), costs of competing interventions (standard therapy, BT, and omalizumab), and utility values associated with each of the health states. These model parameters were elicited from published literature, which are described in detail in the following sections.

**Table 1:** Model Parameters.

Parameters	Value	Probability distribution
**Age at baseline**	40	-
**Rate of exacerbation per person-year**		
*standard therapy*		
Exacerbation requiring oral corticosteroids [10,18,26]	1.35	Log-normal(0.29, 0.10)
Exacerbation requiring emergency room visit [10]	0.07	Log-normal(-2.72, 0.10)
Exacerbation requiring hospitalizations [10]	0.06	Log-normal(-2.79, 0.10)
**Relative rate of exacerbation per person-year (reference is standard therapy)** *		
*BT*		
Exacerbation requiring oral corticosteroids [7,27,36]	0.48	Log-normal(-0.73, 0.09)
Exacerbation requiring emergency room visit [7,28,36]	0.19	Log-normal(-1.64, 0.12)
Exacerbation requiring hospitalizations [7,28,36]	0.30	Log-normal(-1.21, 0.14)
*Omalizumab*		
Exacerbation requiring oral corticosteroids [10,18,26]	0.63	Log-normal(-0.46, 0.01)
Exacerbation requiring emergency room visit [10]	0.40	Log-normal(-0.92, 0.14)
Exacerbation requiring hospitalizations [10]	0.49	Log-normal(-0.72, 0.12)
**Risk of death from hospitalization (30 days)** [41]	0.02	Beta(1.10, 43.22)
**Background mortality rate** [42]	US life tables	None
**Cost (2013-$US)**		
*Treatment costs (per person year)*		
Standard therapy [18,30,31]	$2,610	-
Omalizumab [10,18]	$22,700	-
BT †	$14,900	-
*Other costs(unit cost)* [18,29]		
Exacerbation requiring oral corticosteroids	$130	Γ(100, 0.77)
Exacerbation requiring emergency room visit	$594	Γ(98.01, 0.17)
Exacerbation requiring hospitalizations	$9,900	Γ(100.08, 0.01)
**Health state utility values**		
*Exacerbation-free*		
Standard therapy [18,26,33,35]	0.67	Beta(5.92, 2.93)
Utility difference for omalizumab (reference as standard therapy) [37]*	0.04	N(0.04, 0.004)
Utility difference for BT (reference as standard therapy) [36]*	0.03	N(0.03, 0.02)
*Exacerbation requiring corticosteroids* [18,33,34]	0.57	Beta(0.51, 0.38)
*Exacerbation requiring emergency room visit* [18,34]	0.45	Beta(0.36, 0.45)
*Exacerbation requiring hospitalizations* [18,33,34]	0.33	Beta(0.15, 0.30)

All costs are adjusted to 2013 USA dollars. BT: Bronchial thermoplasty. N(x, y): Normal distribution with mean x and standard deviation y. Γ (x, y): distribution with shape parameter x, and rate parameter y. Beta(x, y): beta distribution with shape1 parameter x, and shape2 parameter y. Log-Normal(x, y): Log-Normal distribution with log-scale parameter x, and shape parameter y.

*: Details in S1 Fig.

†: Calculated based on the costs of three catheters, facility and professional fee, cost associated with possible hospitalization post BT, and cost associated with possible re-scheduling BT. Cost of three catheters, facility, and professional fee was derived from a published study at $14,100 [32]. Cost associated with possible hospitalization post BT was calculated based on 8% chance of hospitalization immediately post BT [7], and $9,900 as a unit cost of hospitalization [18,29] (0.08*$9,900 = $792). Cost associated with possible re-scheduling BT was calculated based on 10% chance of re-scheduling (consultation with an expert clinician in our team), and the unit cost of physician visit (i.e., $66) [30] adjusted to 2013 US dollars using the US consumer price index (0.1*$66 = $6.6).

#### Transition probabilities

There is no study directly comparing exacerbation rates between BT and omalizumab. However, there is indirect evidence through comparisons of BT and omalizumab with standard therapy [7,10,18,26]. We used data from a meta-analysis by Bousquet et al [10] to inform the relative rates (RRs) of exacerbations requiring ED and hospitalization for omalizumab versus standard therapy. The RR of exacerbations requiring OCS for omalizumab versus standard therapy was informed by Campbell et al [18], which itself was based on the studies by Bosuquet et al [10] and Humbert et al [26]. These RRs for exacerbations requiring OCS, ED, and hospitalization were 0.63 (95% credible interval [CrI]: 0.55, 0.73), 0.40 (95% CrI: 0.19–0.82), and 0.49 (95% CrI: 0.25–0.97), respectively.

To determine the RRs of exacerbations for BT versus standard therapy we performed a meta-analysis. For the RR of OCS, results of the AIR [27] and AIR2 [7] trials were pooled. For the RRs of ED and hospitalization, results of the RISA [28] and AIR2 [7] trials were pooled, and a random-effects (RE) meta-analysis framework was adopted. Since there were only a few studies informing the efficacy of BT, for constructing the CrIs around its point estimates, between-study variation was borrowed from a meta-analysis of 7 studies of omalizumab with RR of exacerbations as the outcome [10]. Details of this meta-analysis are explained in the Online Supporting Information (S1). However, in a separate probabilistic sensitivity analysis (PSA), we also reported the cost-effectiveness results based on the original 95% CrIs of BT directly estimated from our RE meta-analysis of BT trials. The pooled RR of exacerbations requiring OCS, ED, and hospitalization from these sources was 0.48 (95% CrI: 0.26, 0.88), 0.19 (95% CrI: 0.10, 0.39), and 0.30 (95% CrI: 0.14, 0.62), respectively. These RRs along with the annual rates of exacerbations in the standard therapy, which were informed from previous studies [10,18,26], were used in the formula, probability = 1-exp(-rate/52), to calculate the weekly transition probabilities from exacerbation-free to the three exacerbation states [23].

#### Costs

All costs were adjusted to 2013 US dollars ($). Cost parameters included costs of standard therapy (controller and reliever medication), BT, omalizumab, and costs of exacerbations stratified by those requiring OCS, ED, and hospitalization. The costs of the three types of exacerbations were derived from previous US studies [18,29].

The costs of standard therapy was calculated based on the published literature [18,30,31]. The costs of omalizumab was based on the number of 150mg-vials and administrations needed per year in patients with moderate-to-severe allergic asthma [10,18].

There is still lack of sufficient evidence around costs of BT as a recent technology. For our model we used an average cost estimate of $14,900 per patient. To derive this value, we used a published study to estimate the average cost of three catheters, facility and professional fee for BT as $14,100 in the US [32], to which we also added the average per patient costs of BT’s possible adverse events (i.e., hospitalization post BT and re-scheduling BT procedure). Details of BT’s costs are represented in ***Table 1***.

#### Health state utility values (HSUVs)

The point estimates and probability distributions assigned to HSUVs are shown in ***Table 1***. HSUVs for exacerbation states were informed from representative publications [18,33,34]. Utilities for exacerbation-free state was derived from the Asthma Quality of Life Questionnaire (AQLQ) in the INNOVATE trial [26], and utilities for exacerbation states were derived from a multi-center UK study on moderate-to-severe asthma [34]. HSUVs associated with exacerbation states were assumed to be the same across different interventions. On the other hand, we allowed the HSUV of the exacerbation-free state to be different between the three interventions, incorporating the potentially distinct impact of these interventions on symptoms and health-related quality of life outside of the period of exacerbations. The HSUV for the exacerbation-free state under standard therapy was the reference value, upon which changes in HSUV associated with BT and omalizumab were modeled. This reference value of 0.67 has been reported by the National Institute for Health and Care Excellence (NICE) [33], which was calculated based on AQLQ domain scores from INNOVATE study [26] and an algorithm by Tsuchiya et al [35] to map AQLQ to EQ-5D utility values. The changes associated with omalizumab and BT were estimated from the changes in AQLQ between the respective treatments and standard therapy from the published studies [36,37]. We used the same validated algorithm by Tsuchiya et al [35] to convert AQLQ to HSUVs. Details of this analysis can be found in the Online Supporting Information (S1). This resulted in the point-estimate HSUV of 0.70 (95% CrI: 0.38, 0.95) and 0.71 (95% CrI: 0.39, 0.96) for BT and omalizumab, respectively. In a sensitivity analysis we varied these values to investigate their impact on the outcomes.

#### Efficacy and adverse events of BT

Even though three studies have already shown the efficacy of BT on reducing the number of exacerbations [7,27,28], there is still substantial uncertainty around BT’s real-world effectiveness and its long-term health benefits [36]. The three studies have generally used similar methodology. In addition, there might be a risk of bias for effectiveness of BT as two of these three studies did not have a sham intervention for the control arm [36]. Furthermore, there are some possible adverse events associated with BT such as requirement for inpatient care after the procedure and need for re-scheduling the procedure in case of asthma symptoms on the procedure day [7]. Castro et al have shown there is 8% chance of hospitalization in the week following BT [7], and since there was no evidence on chance of cancelling and re-scheduling BT in the literature, after a consultation with the expert clinicians of our team we assigned a chance of 10% for this adverse event.

### Analysis

We ran our model separately for standard therapy, BT, and omalizumab, and calculated the average discounted total costs, discounted QALYs, and (undiscounted) number of weeks with exacerbations. The base case results were generated by running the model with the point estimate of parameters.

For PSA, we used a Monte-Carlo simulation with 10,000 iterations by randomly sampling from the distribution of model parameters and calculating the outcomes. Probability distributions were assigned to the model parameters based on the literature or expert opinion (i.e., chance of cancelling and re-scheduling BT). The main outcomes of the PSA were cost-effectiveness plane (CE-Plane) and cost-effectiveness acceptability curve (CEAC). We additionally calculated the expected value of information (EVPI) at different willingness-to-pay (WTP) values to further quantify the extent of uncertainty and the potential value of future research.

We also carried out detailed deterministic sensitivity analyses to evaluate the robustness of the results against variation in the assumptions and definitions. Specifically, given the uncertainties around the costs of BT, we performed a dedicated sensitivity analysis for this parameter, in which we varied the costs of BT from $8,000 to $30,000.

Other sensitivity analyses included varying other treatment costs, costs of exacerbations, time horizon, rates of exacerbations for standard therapy, RRs of exacerbations, and probability of early hospitalization post BT. In the sensitivity analyses that considered a longer time-horizon beyond five years, we also extrapolated the RR of treatments assuming both of the constant and exponentially declining effects after the fifth year. We also repeated the PSA by using the between-study variance estimate for RR of BT from the meta-analysis of BT trials (as opposed to omalizumab trials in the base case analysis). Given the level of precision in the input values for costs, we rounded all the cost estimates from the model to their nearest thousand value.

## Results

***Table 2***documents the main outcomes of the analysis. Over five years, for standard therapy, the average discounted costs, QALYs, number of exacerbations, and proportion of the population who died were $15,400, 3.08, 7.00, and 0.01, respectively. The corresponding values for BT were $28,100, 3.24, 3.31, and 0.01, and for omalizumab they were $117,000, 3.26, 4.39, and 0.01. Our results indicate that omalizumab was the most effective therapy in terms of QALYs gained. Relative to standard therapy, treatment with BT was associated with an ICER of $78,700/QALY, and treatment with omalizumab was associated with an ICER of $552,000/QALY.

**Table 2:** The expected value and 95% CrI of outcomes over a five year time frame.

Outcome	Standard therapy	BT	omalizumab
**Cost (95% CrI)**	$15,400 ($14,700-$16,300)	$28,100 ($27,600-$29,100)	$117,000 ($116,000-$118,000)
**QALYs (95% CrI)**	3.08(1.64–4.21)	3.24(1.78–4.38)	3.26(1.80–4.40)
**Number of oral corticosteroid courses (95% CrI)**	6.40(5.27–7.64)	3.15(1.71–5.77)	4.12(3.25–5.14)
**Number of emergency department visits (95% CrI)**	0.31(0.26–0.38)	0.06(0.03–0.13)	0.13(0.06–0.27)
**Number of hospitalizations (95% CrI)**	0.30(0.24–0.36)	0.09(0.04–0.18)	0.15(0.07–0.30)
**Proportion of population died (95% CrI)**	0.012(0.010–0.016)	0.011(0.010–0.012)	0.011(0.010–0.014)
**ICER**			
*BT versus standard therapy*	Reference	$78,700/QALY	-
*Omalizumab versus BT*	-	Reference	$3.86 million/QALY
*Omalizumab versus standard therapy*	Reference	-	$552,000/QALY

BT: bronchial thermoplasty, CrI: credible interval, QALY: quality-adjusted life year, ICER: Incremental cost-effectiveness ratio.

In the life time analysis that assumed an exponentially declining effect for BT after the 5^th^ year, the ICER of BT vs. standard therapy, omalizumab vs. BT, and omalizumab vs. standard therapy was $12,500/QALY, $3.15 million/QALY, and $529,000/QALY, respectively.

**Fig 2:**
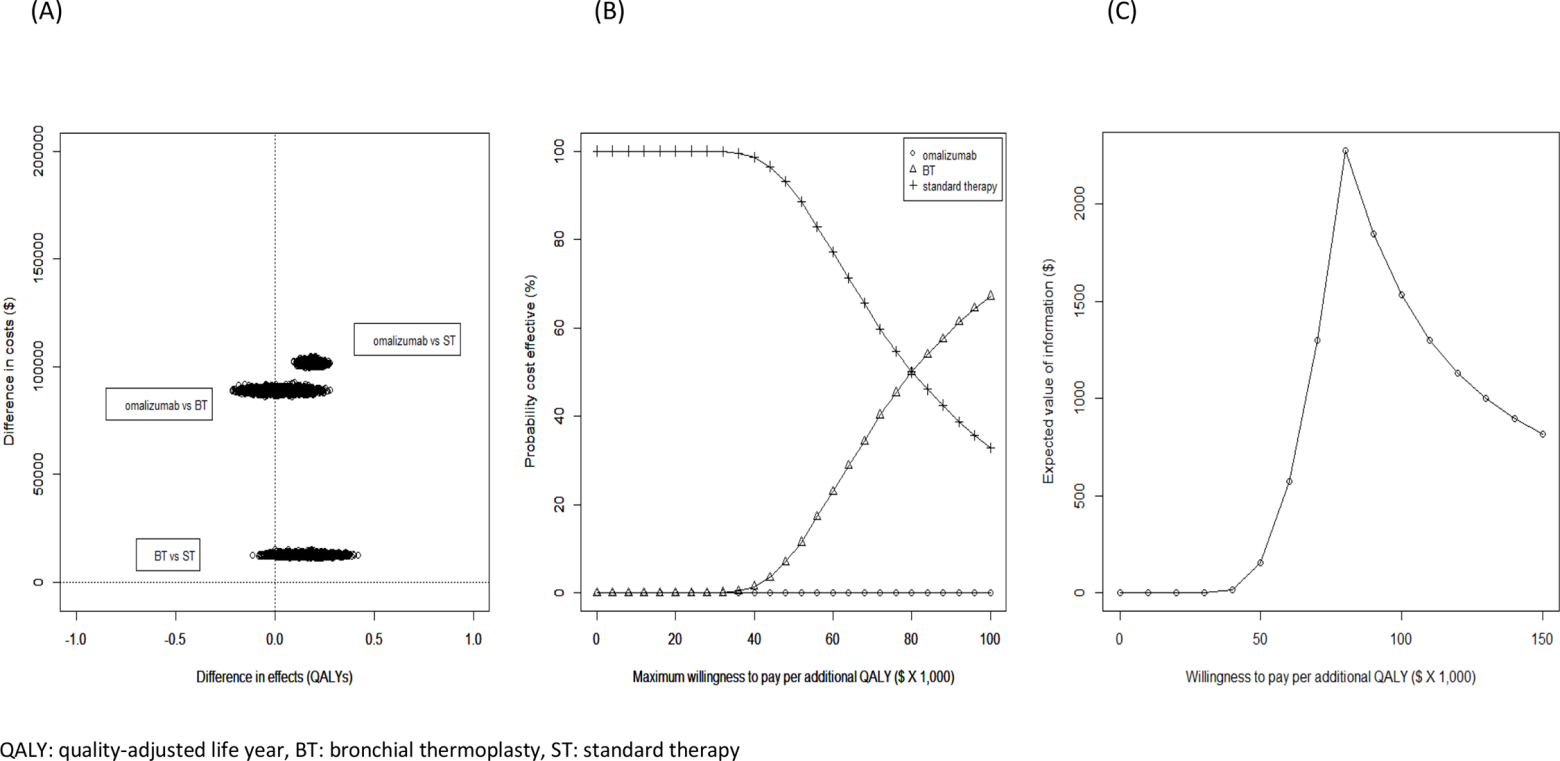
(A) Cost-effectiveness plane; (B) Cost-effectiveness acceptability curve; and (C) Expected value of information.

***Fig 2***shows the results of PSA. The CE-planes for BT versus standard therapy, omalizumab versus BT, and omalizumab versus standard therapy are shown in Fig 2(A). Overall, there was substantial uncertainty around comparisons that involved BT. While in the majority of the simulation runs BT was more effective than standard therapy, overall it was associated with higher costs. Also, there was little uncertainty about BT being cost-saving compared with omalizumab, but omalizumab was associated with consistently higher gain in QALYs.

Fig 2(B) shows the CEAC, which indicates the probability of BT being cost-effective was 9% at the WTP of $50,000/QALY and 67% at the WTP of $100,000/QALY. The EVPI at different WTP values is presented in Fig 2(C). EVPI at the WTP of $50,000/QALY was $155 per individual, which increased to $1,530 per individual at the WTP of $100,000/QALY.

**Fig 3:**
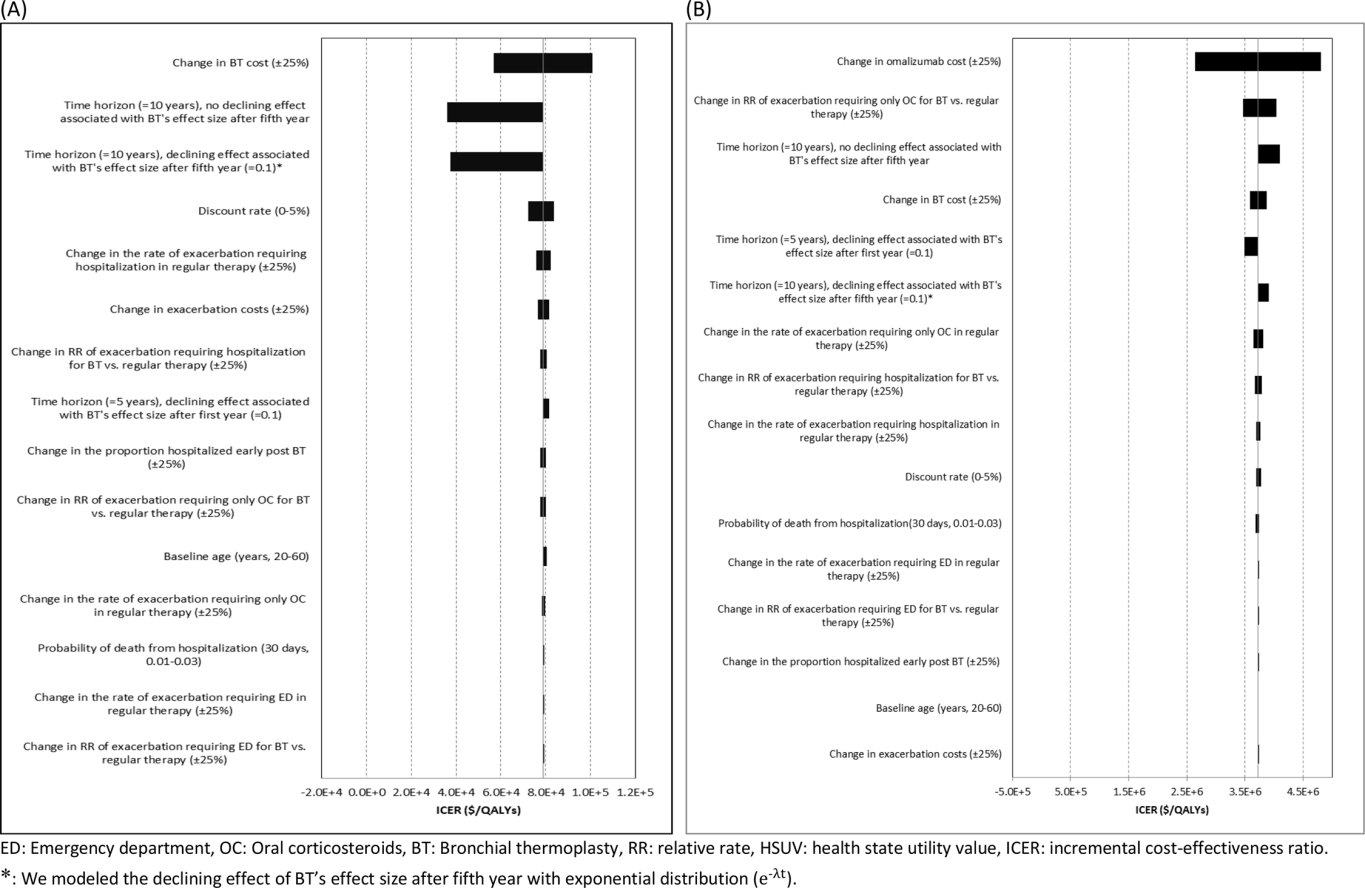
One-way sensitivity analysis: (A) BT versus standard therapy, (B) omalizumab versus BT.

***Fig 3***represents the results of sensitivity analyses, with panel A for BT versus standard therapy and panel B for omalizumab versus BT. As seen in both panels, costs of omalizumab and BT had the most pronounced impact on ICERs. Decreasing the costs of BT and omalizumab by 25% reduced the ICER of BT relative to standard therapy by 28% (to $57,000/QALY), and ICER of omalizumab relative to BT by 29% (to $2.65 million/QALY), respectively. Other sensitivity analyses demonstrated that results were particularly sensitive to the utility of exacerbation-free state for omalizumab and BT. Changing the utility difference between omalizumab and standard therapy from 0.03 to 0.05 (derived based on a Cochrane review on omalizumab [37]) changed the ICER of omalizumab relative to BT from -$5.21 million/QALY (BT being dominant) to $1.20 million/QALY. Also, changing the utility difference between BT and standard therapy from 0 (i.e., no change) to 0.06 (based on a Cochrane review on BT [36]) changed the ICER of BT relative to standard therapy from $1.31 million/QALY to $44,700/QALY. In addition, a separate PSA, in which the original CrIs for RRs of exacerbations for BT directly estimated from our meta-analysis were used (instead of using the borrowed between-study variation from omalizumab studies), did not change the cost-effectiveness results. In this scenario, the probability of BT being cost-effective vs. omalizumab and standard therapy remained the same as the base case, 9% at the WTP of $50,000/QALY, and 67% at the WTP of $100,000/QALY.

**Fig 4:**
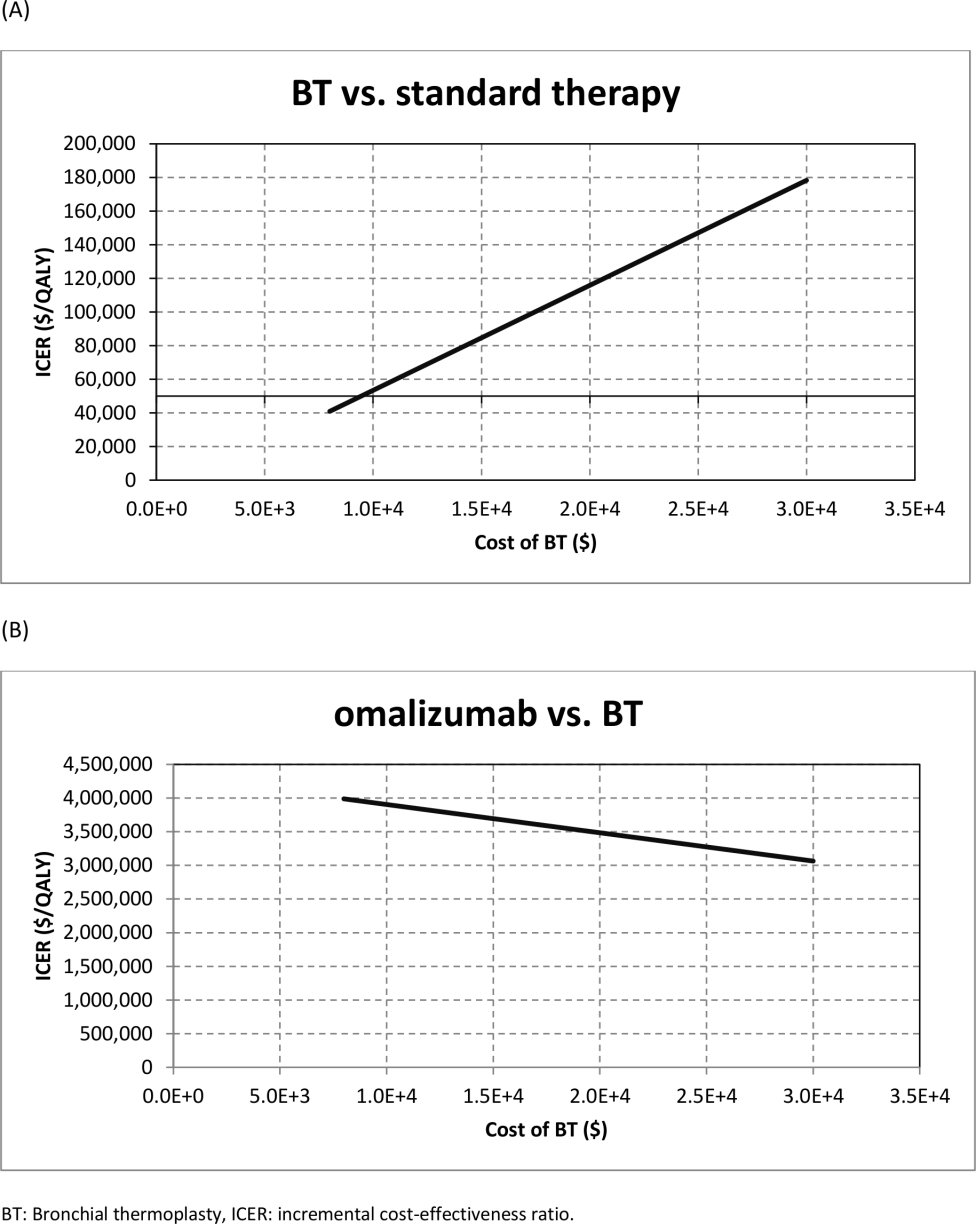
Sensitivity analysis for the costs of BT. Incremental cost-effectiveness ratio as a function of BT’s cost: (A) BT versus standard therapy, (B) omalizumab versus BT.

***Fig 4***shows the results of a sensitivity analysis on the costs of BT. In this analysis, we varied the costs of BT from $8,000 to $30,000 per patient and calculated the ICER for BT relative to standard therapy as well as omalizumab relative to BT. Changing the costs of BT from $8,000 to $30,000 increased the ICER of BT relative to standard therapy from $40,900/QALY to $178,000/QALY, and decreased the ICER of omalizumab relative to BT from $3.99 million/QALY to $3.06 million/QALY. The threshold value for the costs of BT that result in the ICER of BT versus standard therapy being $50,000 and $100,000 was approximately $9,000 and $17,000, respectively.

## Discussion

In this study we compared the cost-effectiveness of standard therapy, BT, and omalizumab for the management of moderate-to-severe allergic asthma. Although there is still significant uncertainty around BT efficacy, based on available evidence, our study suggests that BT can be a cost-effective option relative to the other two comparative treatments if the policy makers are willing to pay more than $80,000/QALY. However, our study also indicated the presence of substantial uncertainty in the results. The chance of BT being cost-effective compared with omalizumab and standard therapy was 67% at the WTP of $100,000/QALY. We also showed that in the target population of this study, omalizumab was not cost-effective compared with standard therapy despite being associated with significant clinical improvements. This finding is consistent with the majority of previous evaluations [17–23]. We also developed a freely-accessible web application (available from http://resp.med.ubc.ca/software/ipress/bt-cea/), which provides an interactive framework for users to investigate the results as a function of different input values.

There are some limitations in our study. The effect of treatment on rates of exacerbations and hospitalizations were based on short-term clinical trials [10,18,26], but were assumed to persist over the 5-year time horizon of the study. Nevertheless, there is evidence to support our assumption, which has shown the consistent effect of BT over five years [9]. In addition, there is scarce evidence on the optimal duration of omalizumab therapy as in studies of omalizumab, the dosage has often been reduced or the drug has been completely withdrawn in some subjects. As the primary outcome of studies evaluating BT was asthma exacerbations, we constructed our model around asthma exacerbations. Our model’s health states were similar to the asthma policy model’s states [38] and other previously published evaluations [17–23]. Nevertheless, this choice of model precluded us from investigating the effect of interventions on levels of asthma control defined by the guidelines such as GINA [11]. It is worth noting that we indirectly considered the effect of treatments on symptom control by incorporating differential impact of treatment on quality of life associated with exacerbation-free health states. Future studies are needed to investigate the effect of BT on transitioning among levels of control as well as the impact of BT on quality of life aside from its effect on the rate of exacerbations as potentially important parameters determining its cost-effectiveness. In addition, in this study we might have underestimated the uncertainty for HSUVs by converting the AQLQ scores to HSUVs [39]; however, in the absence of direct evidence on the final outcome of interest, using intermediate outcomes is a reasonable alternative. We also minimized the risk of bias by applying the same validated mapping algorithm technique [35] to both BT and omalizumab to calculate their impact on HSUVs. For our estimates of treatment effect, we had to rely on existing available evidence from a few BT studies [7,8,27], but due to concerns about homogeneity in the design and included populations of the published studies, we used evidence from omalizumab trials to estimate the between-study variability in the main analysis. There might also be a risk of bias in the point estimate of treatment effect as two [8,27] of these three studies did not include a sham intervention in the control group [36]. In addition, the relatively large placebo effect in the sham arms of BT trials might suggest some patients did not receive optimal treatments before entering to the study, making the observed effect of BT less relevant to the context of this evaluation which considers BT after maximum dose of double therapy has failed to achieve asthma control.

Omalizumab is often used for moderate-to-severe asthma patients with increased level of IgEs. Since no study has exclusively studied the effect of BT on allergic asthma patients, we had to assume that BT confers the same benefit to allergic asthma patients as to the general asthma population. Since some studies of BT had only recruited patients with moderate asthma, using such estimates for the treatment effects in moderate-to-severe asthma is an extrapolation. As for the costs, similar to any other emerging technology, there is lack of evidence around the true costs of BT. It is likely that widespread implementation of BT might result in different costs profiles than those estimated from a few centers investigating this technology. However, to ensure the comprehensiveness of our evaluation, we re-assessed the cost-effectiveness of BT in a separate and dedicated sensitivity analysis, in which we varied the costs of BT by a wide margin. Finally, given the invasive nature of BT and its short-term adverse events, it is important to understand that with more widespread dissemination of the technology, operator skills will become an important consideration. The current body of evidence for the efficacy of BT is primarily based on studies done in more specialized centers with an interest in moderate-to-severe asthma with skilled operators. However, it is conceivable that with less skilled technicians, the outcomes and adverse events associated with BT may be worse than those from clinical trials.

Uncontrolled asthma is associated with significant economic and humanistic burden [40]. Given the current therapies and the likely arrival of further expensive monoclonal antibody treatments for severe asthma, it will be important for clinicians and policy makers to develop a framework by which these health technologies can be formally assessed in terms of both costs and health outcomes. This study addresses the cost-effectiveness of BT compared to the currently only available monoclonal antibody for asthma, as well as to standard therapy, based on the currently available evidence. The framework adopted in this study can be used as a resource to inform policy makers and health-care providers on the benefits of these interventions. Our overall conclusion is that there is a clear need for further comparative studies of the health technologies assessed in the current project.

## Supporting Information

S1 Fig Additional descriptions for estimating model parameters. (DOCX)

## Abbreviations

ICER: incremental cost-effectiveness ratio
QALY: quality-adjusted life years
WTP: willingness to pay
ICS: inhaled corticosteroids
LABA: long-acting beta agonists
GINA: Global Initiative for Asthma
HSUV: health state utility value
CEAC: cost-effectiveness acceptability curve
RCT: randomized controlled trial
CrI: credible interval
RR: relative rate
