# Supplementary material for: Cost-Effectiveness of Bronchial Thermoplasty, Omalizumab, and Standard Therapy for Moderate-to-Severe Allergic Asthma
Source: PLoS One. 2016 Jan 11;11(1):e0146003. doi: 10.1371/journal.pone.0146003 (PMC4709059; doi:10.1371/journal.pone.0146003)
Supplement: S1 Fig — (DOCX) [file pone.0146003.s001.docx]

Supporting Information

**S1 Fig. Additional descriptions for estimating model parameters**

**1-Relative rate of exacerbation for bronchial thermoplasty (BT) relative to standard therapy**

We performed a meta-analysis of the two primary published studies to estimate the relative rate (RR) of exacerbation (including exacerbation requiring oral corticosteroids (OCS), emergency department visit (ED), and hospitalizations) for BT relative to standard therapy. For estimating the RR of OCS we pooled data from studies by Cox et al [1] (AIR trial), and Castro et al [2] (AIR2 trial) using random effects models. For estimating the RR of ED and hospitalization we pooled data from studies by Pavord et al [3] (RISA trial) and Castro et al [2] (AIR2 trial). Forest plots of these meta-analyses are shown in the S1 Fig.

**S1 Fig. Forest plot for RR of exacerbation requiring (A) OCS; (B) ED visit; (C) hospitalization.**

(A)


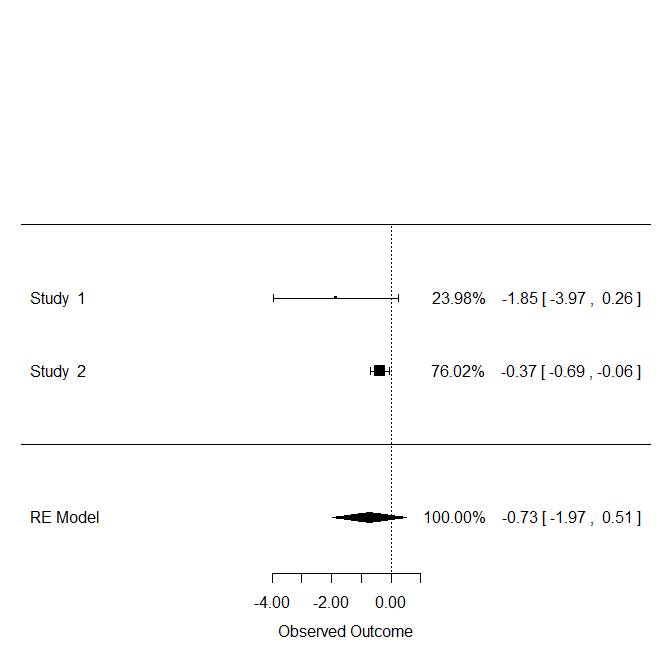


(B)


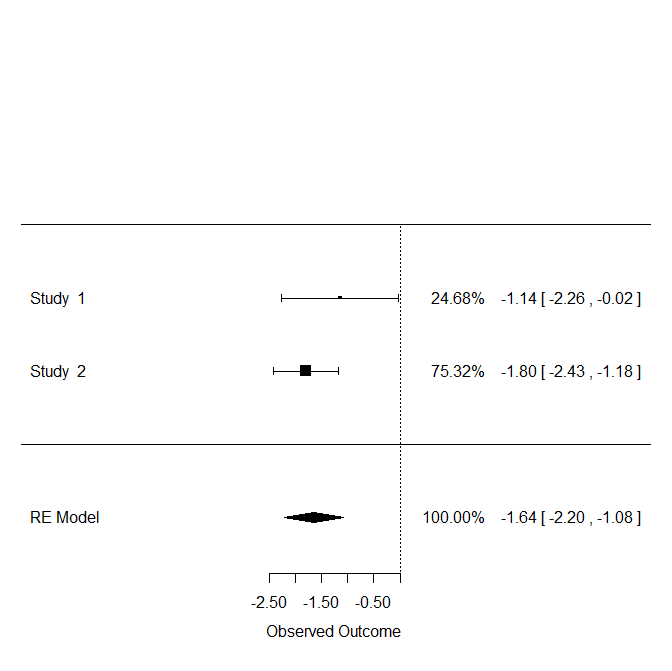


(C)


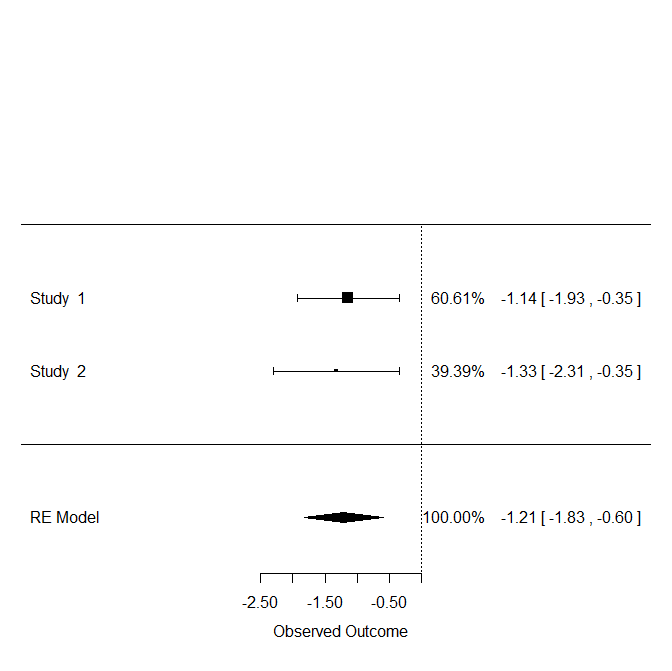
 RR: relative rate, OCS: oral corticosteroids, ED: emergency department. For panel A study 1 is by Cox et al [1] , and study 2 is by Castro et al [2] . For panel B and C study 1 is by Pavord et al [3] , and study 2 is by Castro et al [2].

The pooled RR of exacerbation requiring OCS, ED visit, and hospitalization from these sources was 0.482 (95% Credible interval [CrI]: 0.264, 0.881), 0.194 (95% CrI: 0.098, 0.385), and 0.297 (95% CrI: 0.144, 0.616), respectively. Because of the lack of evidence on BT compared to omalizumab, for calculating the 95% credible interval, between-study variation was borrowed from a meta-analysis of omalizumab studies [4]. Meta-analysis was performed in statistical software R 3.1.0 [5]. We used log-Normal distribution to make these parameters probabilistic.

**2-Health state utility value (HSUV) for exacerbation-free state**

For calculating the HSUV associated with exacerbation-free state in BT and omalizumab, we used published meta-analyses [6,7]. HSUV associated with exacerbation-free state in standard therapy was derived from [8–11]. Also HSUVs for exacerbation states (requiring OCS; ED visit; and hospitalization) were derived from [8,11,12]. Torrego et al [6] have reported the difference of Asthma Quality of Life Questionnaire (AQLQ) scores for BT versus standard therapy at 12-month of follow-up . First, we converted AQLQ to EQ-5D using a validated algorithm by Tsuchiya et al [10]. Second, we ran our model for both BT and standard therapy over a period of 12-month to get the proportion of patients in each of the heath states at the 12^th^ month, based on which HSUV of exacerbation-free state for BT was calculated.

Similarly for calculating the HSUV of exacerbation-free state in omalizumab, we used the reported meta-analysis on AQLQ scores for omalizumab relative to standard therapy by Rodrigo et al [7], and repeated the same procedure as explained above. Since the follow-up time was not reported by Rodrigo et al [7], we used the average follow-up times of all studies included in their meta-analysis (follow-up time=16 weeks in studies by Busse et al [13], Soler et al [14], and Holgate et al [15]; follow-up time=28 weeks in studies by Vignola et al [16], and Humbert et al [9]).

# References for Supporting Information

1. Cox G, Thomson NC, Rubin AS, Niven RM, Corris PA, Siersted HC, et al. Asthma control during the year after bronchial thermoplasty. N Engl J Med. 2007 Mar 29;356(13):1327–37.

2. Castro M, Rubin AS, Laviolette M, Fiterman J, Lima MDA, Shah PL, et al. Effectiveness and Safety of Bronchial Thermoplasty in the Treatment of Severe Asthma A Multicenter, Randomized, Double-Blind, Sham-Controlled Clinical Trial. Am J Respir Crit Care Med. 2010 Jan 15;181(2):116–24.

3. Pavord ID, Thomson NC, Niven RM, Corris PA, Chung KF, Cox G, et al. Safety of bronchial thermoplasty in patients with severe refractory asthma. Ann Allergy Asthma Immunol. 2013 Nov;111(5):402–7.

4. Bousquet J, Cabrera P, Berkman N, Buhl R, Holgate S, Wenzel S, et al. The effect of treatment with omalizumab, an anti-IgE antibody, on asthma exacerbations and emergency medical visits in patients with severe persistent asthma. Allergy. 2005 Mar;60(3):302–8.

5. R Development Core Team. R: A Language and Environment for Statistical Computing [Internet]. Vienna, Austria: R Foundation for Statistical Computing; 2011 [cited 2009 Nov 16]. Available from: http://www.r-project.org/.

6. Torrego A, Solà I, Munoz AM, Roqué I Figuls M, Yepes-Nuñez JJ, Alonso-Coello P, et al. Bronchial thermoplasty for moderate or severe persistent asthma in adults. Cochrane Database Syst Rev. 2014;3:CD009910.

7. Rodrigo GJ, Neffen H, Castro-Rodriguez JA. Efficacy and safety of subcutaneous omalizumab vs placebo as add-on therapy to corticosteroids for children and adults with asthma: A systematic review. CHEST J. 2011 Jan 1;139(1):28–35.

8. National Institute for Health and Care Excellence (NICE). Omalizumab for treating severe persistent allergic asthma (review of technology appraisal guidance 133 and 201). 2013. [Internet]. [cited 2014 Jun 2]. Available from: http://www.nice.org.uk/nicemedia/live/13550/61399/61399.pdf

9. Humbert M, Beasley R, Ayres J, Slavin R, Hébert J, Bousquet J, et al. Benefits of omalizumab as add-on therapy in patients with severe persistent asthma who are inadequately controlled despite best available therapy (GINA 2002 step 4 treatment): INNOVATE. Allergy. 2005 Mar;60(3):309–16.

10. Tsuchiya A, Brazier J, McColl E, Parkin D. Deriving preference-based single indices from non-preference based condition-specific instruments: converting AQLQ into EQ5D indices [Internet]. University Library of Munich, Germany; 2002 [cited 2013 Aug 8]. Report No.: 29740. Available from: http://ideas.repec.org/p/pra/mprapa/29740.html

11. Campbell JD, Spackman DE, Sullivan SD. The costs and consequences of omalizumab in uncontrolled asthma from a USA payer perspective. Allergy. 2010 Sep;65(9):1141–8.

12. Price D, Brown RE, Lloyd A. Burden of poorly controlled asthma for patients and society in the UK. Prim Care Respir J 2004;13:113.

13. Busse W, Corren J, Lanier BQ, McAlary M, Fowler-Taylor A, Cioppa GD, et al. Omalizumab, anti-IgE recombinant humanized monoclonal antibody, for the treatment of severe allergic asthma. J Allergy Clin Immunol. 2001 Aug;108(2):184–90.

14. Solèr M, Matz J, Townley R, Buhl R, O’Brien J, Fox H, et al. The anti-IgE antibody omalizumab reduces exacerbations and steroid requirement in allergic asthmatics. Eur Respir J. 2001 Aug;18(2):254–61.

15. Holgate ST, Chuchalin AG, Hébert J, Lötvall J, Persson GB, Chung KF, et al. Efficacy and safety of a recombinant anti-immunoglobulin E antibody (omalizumab) in severe allergic asthma. Clin Exp Allergy J Br Soc Allergy Clin Immunol. 2004 Apr;34(4):632–8.

16. Vignola AM, Humbert M, Bousquet J, Boulet L-P, Hedgecock S, Blogg M, et al. Efficacy and tolerability of anti-immunoglobulin E therapy with omalizumab in patients with concomitant allergic asthma and persistent allergic rhinitis: SOLAR. Allergy. 2004 Jul;59(7):709–17.
